# Diversity of antibiotic-resistance genes in Canadian isolates of *Aeromonas salmonicida* subsp. *salmonicida*: dominance of pSN254b and discovery of pAsa8

**DOI:** 10.1038/srep35617

**Published:** 2016-10-18

**Authors:** Mélanie V. Trudel, Antony T. Vincent, Sabrina A. Attéré, Myriam Labbé, Nicolas Derome, Alexander I. Culley, Steve J. Charette

**Affiliations:** 1 Institut de Biologie Intégrative et des Systèmes (IBIS), Université Laval, Quebec City, QC, G1V 0A6, Canada; 2 Département de biochimie, de microbiologie et de bio-informatique, Faculté des sciences et de génie, Université Laval, Quebec City, QC, G1V 0A6, Canada; 3 Centre de recherche de l’Institut universitaire de cardiologie et de pneumologie de Québec (IUCPQ), Quebec City, QC, G1V 4G5, Canada; 4 Groupe de Recherche en Écologie Buccale (GREB), Faculté de médecine dentaire, Université Laval, Quebec City, QC, G1V 0A6, Canada; 5 Département de biologie, Faculté des sciences et de génie, Université Laval, Quebec City, QC, G1V 0A6, Canada

## Abstract

The bacterium *Aeromonas salmonicida* subsp. *salmonicida* is a common pathogen in fish farms worldwide. Since the antibiotic resistance of this bacterial species is on the increase, it is important to have a broader view on this issue. In the present study, we tested the presence of known plasmids conferring multi-drug resistance as well as antibiotic resistance genes by a PCR approach in 100 Canadian *A. salmonicida* subsp. *salmonicida* isolates. Our study highlighted the dominance of the conjugative pSN254b plasmid, which confers multi-drug resistance. We also identified a new multi-drug plasmid named pAsa8, which has been characterized by a combination of sequencing technologies (Illumina and Oxford nanopore). This new plasmid harbors a complex class 1 integron similar to the one of the *Salmonella genomic island 1* (SGI1) found in *Salmonella enterica* and *Proteus mirabilis*. Consequently, in addition to providing an update on the *A. salmonicida* subsp. *salmonicida* isolates that are resistant to antibiotics, our data suggest that this bacterium is potentially an important reservoir of drug resistance genes and should consequently be monitored more extensively. In addition, we describe a screening method that has the potential to become a diagnostic tool that is complementary to other methods currently in use.

The Gram-negative bacterium *Aeromonas salmonicida* subsp. *salmonicida* is a fish pathogen that causes furunculosis worldwide^1^,^2^. In Quebec (Canada), furunculosis is the most common infection encountered in fish farms, especially brook trout farms, and causes between 30 and 60% of reported outbreaks every year^3^. Antibiotics are the most widely used treatment for furunculosis. In Canada, four antibiotics (oxytetracycline, florfenicol [a chloramphenicol analog], sulfadimethoxine/ormetoprim, and sulfadiazine/trimethoprim) are approved by the Veterinary Drugs Directorate (VDD) of Health Canada to treat infected fish. In Quebec, florfenicol is the antibiotic of choice because of its short withdrawal period (12 days) compared to antibiotics such as oxytetracycline and sulfadimethoxine/ormetoprim (42 days)^3^. However, intensive use of these four antibiotics has been correlated with growing number of antibiotic-resistant isolates^3^.

Antibiotic resistance genes in *A. salmonicida* subsp. *salmonicida* are mostly located on plasmids^4^. Among them, pSN254b and pAB5S9b plasmids, which bear multi-drug resistance genes, as well as variants of pRAS3 have been recently described in isolates from Canada^5^. To date, pSN254b has been identified in isolates from Quebec, New Brunswick (Canada) and Nova Scotia (Canada), while pAB5S9b and pRAS3 variants have been identified only in New Brunswick. pAB5S9b and pSN254b bear genes coding for resistance to the three antibiotics used in veterinary medicine in Quebec. There are currently no government-approved antibiotics that are effective against bacterial isolates that host either one of these two plasmids. For their part, pRAS3 variants contain a gene coding for tetracycline resistance^5^. In addition to these plasmids conferring drug resistance (R-plasmids), many others were found in *A. salmonicida*, some of them having a large-panel of genes causing resistance to antibiotics (Table 1).

Even if the plasmidome (i.e., the total plasmid content) of *A. salmonicida* subsp. *salmonicida* harbor multiple R-plasmids, the dispersion of these plasmids and the gravity of the situation are presently unknown. In order to determine the fraction of isolates bearing antibiotic-resistance genes and to infer the geographic distribution of the R-plasmids, we investigated a collection of 100 *A. salmonicida* subsp. *salmonicida* Canadian isolates, using, among other methods, optimized multiplex PCR assays. This approach was also done to assess the presence of new R-plasmids.

This screening showed that pSN254b is the most common R-plasmid in tested Canadian isolates of *A. salmonicida* subsp. *salmonicida*. A new R-plasmid, pAsa8, was also found, which had genetic features that provided additional evidence that *A. salmonicida* subsp. *salmonicida* is an important reservoir for mobile genetic elements as well as antibiotic resistance genes.

## Results

### Plasmid content determined by PCR genotyping

The presence of plasmids containing known antibiotic resistance genes (pAsa4, pAB5S9b, pRAS3.3, pSN254b) has been tested in 100 Canadian *A. salmonicida* subsp. *salmonicida* isolates^5^ using the primers listed in Supplementary Table S1. The results of the genotyping for the 100 isolates are given in Supplementary Table S2. The pSN254b plasmid was the most prevalent (25/100), while the other plasmids were detected in two to four isolates each.

### Multiplex PCR to detect antibiotic resistance genes

We subsequently developed four multiplex PCR assays (see the Methods section) to detect the antibiotic resistance genes known to be present in *A. salmonicida* subsp. *salmonicida* isolates: *floR*, *cat*, *sul1*, *sul2*, *tetA*, *tet*(C), *tet*(E), and *tet*(H). The results of these tests for the 100 *A. salmonicida* subsp. *salmonicida* isolates are presented in Supplementary Table S2.

The *floR* gene was detected in 29 of the 100 isolates, while the *cat* gene was not detected in any of the isolates excepted in A449, which was the positive control. The *floR* gene, which has been detected in isolates with the pSN254b and pAB5S9b plasmids^5^, was also detected in the M15448-11 and M16474-11 isolates, despite the fact that they do not host either of the two plasmids. This result suggests that an unknown genomic entity might be responsible for the presence of the *floR* gene in these two isolates.

The *sul1* and *sul2* genes were detected in 30 and 28 isolates, respectively. Both genes were detected in isolates that contained pSN254b, while only *sul2* was detected in isolates carrying pAB5S9b, as reported previously^5^. The *sul1* gene was detected in four isolates (three from Quebec and one from Ontario (Canada)) with a pAsa4-like plasmid. This multiplex PCR experiment also revealed that the *sul1* gene was present in M15448-11 and M16474-11.

The *tet*(C) gene was detected in four isolates with the pRAS3 plasmid while *tet*(H) was detected in two isolates harboring the pAB5S9b plasmid^5^. The *tet*(E) gene was present in four isolates with a pAsa4-like plasmid. The *tetA* gene was detected in isolates with the pSN254b plasmid^5^ as well as in the M15448-11 and M16474-11 isolates.

### Characterization of pAsa8

The presence of multiple closely related antibiotic resistance genes in M15448-11 and M16474-11 isolates without the detection of any recognized plasmids in these isolates prompted us to sequence the complete DNA of M16474-11 by Illumina and Oxford nanopore (MinION) technologies. Only the DNA of the M16474-11 isolate was sequenced since both isolates (i.e., M15448-11 and M16474-11) exhibited the same profile.

**Figure 1:**
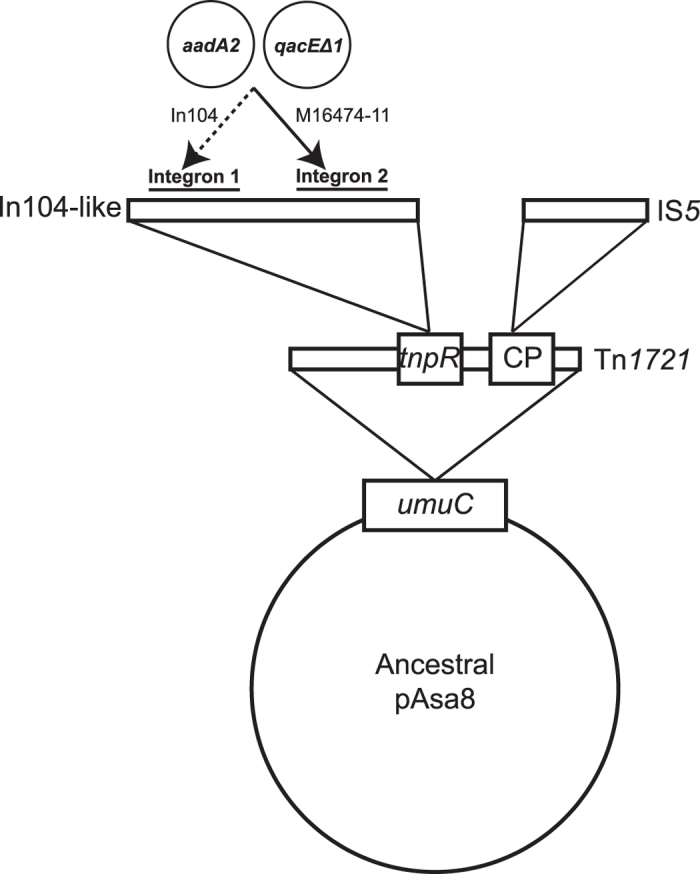
Putative evolutionary scenario of the multidrug region of pAsa8. Only the elements involved are shown: the gene *umuC* of ancestral pAsa8, the genes *tnpR* and the one encoding for the chemotaxis protein (CP) located on the transposon Tn*1721*, the insertion sequence IS*5* and finally the integron In104-like. Cassettes *aadA2* and *qacEΔ1* are located within integron 1 of In104 (dashed arrow), and also within integron 2 of In104-like found on pAsa8 (solid line arrow) (isolate M16474-11).

The sequencing revealed the presence of a new plasmid, that we named pAsa8, of 110 577 bp and which has a region containing many drug resistance genes. This region is the product of multiple integration events involving mobile genetic elements (Fig. 1). One of them is a transposon Tn*1721*^6^, which is integrated in the gene *umuC*, encoding for a UV mutagenesis and repair protein, of the ancestral pAsa8. This transposon brings a *tetA* gene, causing resistance to tetracycline, which explains the positive result of the multiplex PCR.

**Figure 2:**
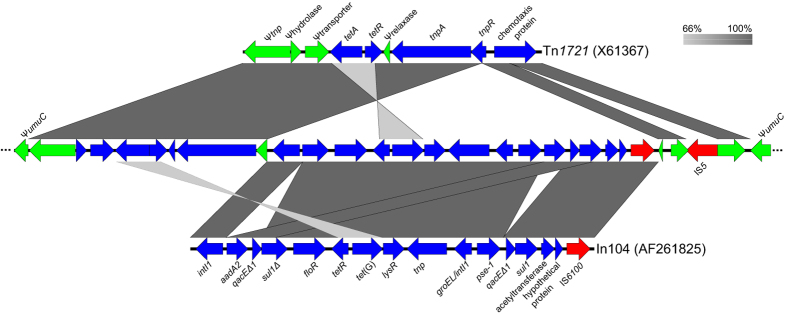
Comparisons of the multi-drug region of pAsa8 with the transposon Tn*1721* and the integron In104 found in SGI.

A second integration event involved a complex class 1 integron In104-like (In4-group) previously found in a 43-kb integrated element known as the *Salmonella genomic island 1* (SGI1) found in *Salmonella enterica*^7^ and *Proteus mirabilis*^8^,^9^,^10^,^11^ and for which numerous variants were found^12^. The In104-like integron allows resistance to florfenicol/chloramphenicol (*floR*), tetracyclines (*tet*(G)), sulfonamides (*sul1*), ampicillin/carbenicillin (bla_PSE-1_) and streptomycin/spectinomycin (*aadA2*) (Fig. 2) in the previously described M16474-11 *A. salmonicida* subsp. *salmonicida* isolate. The complex In104 integron contains two functional *IntI1* integron regions^12^,^13^ with many variants reported^14^. The integron regions found in the In104-like of *A. salmonicida* differs from those of the original In104 found in *S. enterica* serovar *Typhimurium* DT104 in the sense that the cassettes *aadA2* and *qacEΔ1* found in the first integron (*IntI1*) in *S. enterica* are in the second (*groEL/IntI1*) for *A. salmonicida* subsp. *salmonicida* (Fig. 2). The evolutionary scenario that would account for the cassette swap is not clear, but a BLASTn revealed that the second integron region (*groEL/IntI1*) is identical to one of the transposon Tn*2610*, which is formed by parts of Tn*1721*, Tn*21* and SGI1^15^. The third and last integration event involves the integration of an IS*5* into the gene that encodes the chemotaxis protein of Tn*1721*.

Interestingly, the IncU plasmid pRAS1, found in both typical and atypical *A. salmonicida*, was also reported to harbours a Tn*1721* and an integron of the In4 family^16^,^17^. Unfortunately, this plasmid is not sequenced and consequently it would be perilous to postulate any evolutionary links between pRAS1 and pAsa8. As recently reviewed elsewhere, Tn*1721* is present in several strains of the genus *Aeromonas*^4^.

The pAsa8 backbone contains several genes encoding for hypothetical proteins and also has a region containing genes promoting conjugative transfers. However, many of these ORFs are likely pseudogenes or are highly derived relative to sequences in the GenBank nr/nt database (based on BLASTp). No type 2 toxin-antitoxin system was found by TAfinder. A BLASTn query of the nr/nt database with the pAsa8 sequence only gave partial alignments (the multidrug region). However, a BLASTn analysis against the Whole Genome Shotgun (WGS) database (Gammaproteobacteria, taxid:1236) resulted in significant homology to the draft genome of *Aeromonas rivuli* strain DSM 22539 (GenBank: CDBJ00000000.1). This sequence was a result of a recent large-scale study investigating Aeromonad taxonomy and was not extensively analyzed^18^. Interestingly, it was possible to map five contigs from the draft genome of *A. rivuli* on the sequence of pAsa8 using locally CONTIguator version 2.7.4^19^. The five contigs covered almost all of the pAsa8 sequence, with the exception of the region containing the Tn*1721* and the In104-like regions in pAsa8, thus indicating that a similar plasmid, without the integration of these mobile elements, could be present in the strain DSM 22539 of *A. rivuli*. A search for Tn*1721* and In104-like in the complete draft genome of *A. rivuli* was negative.

**Figure 3:**
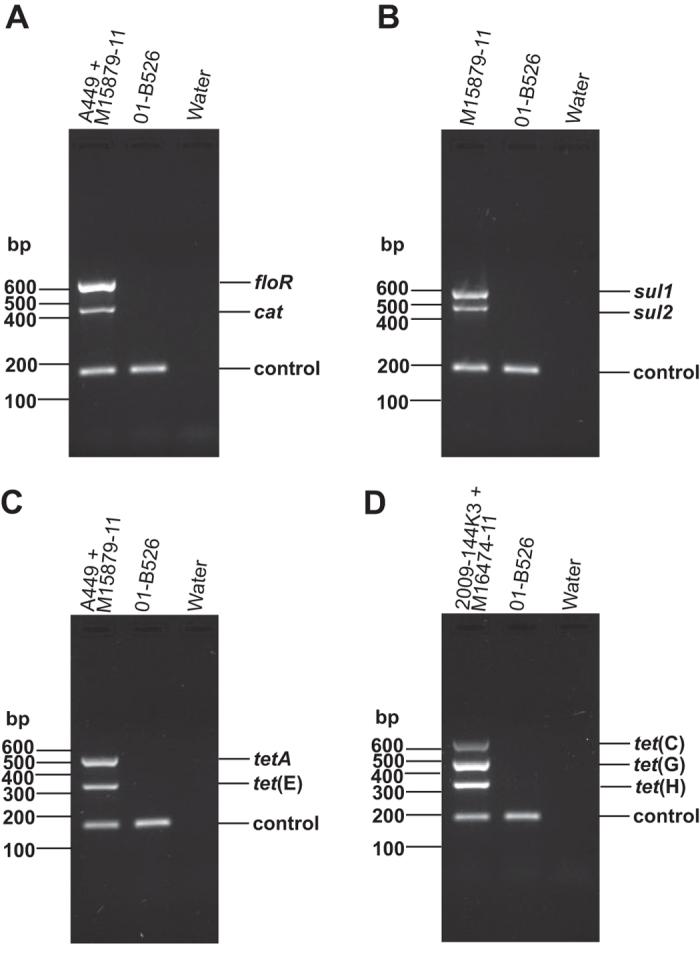
Multiplex PCR targeting genes coding for resistance to chloramphenicol/florfenicol (**A**), sulfonamides (**B**), and tetracyclines (**C**,**D**). Electrophoresis gels with amplicons generated using DNA isolated from positive controls (A449 + M15879-11 in (**A,C**), M15879-11 in B, 2009-144K3 + M16474-11 in D) and from a negative control (01-B526). A specific control for *A. salmonicida* subsp. *salmonicida* using a primer pair that amplified an element of the prophage 1 was included in each multiplex PCR reaction. The amplicon of this product can be seen at the bottom of each gel (control). (**A**) The target *cat* (448 bp) and *floR* genes (632 bp) and (**B**) the *sul2* (449 bp) and *sul1* (550 bp) genes were detected when present. (**C**) Amplicons for *tet*(E) (351 bp) and *tetA* (526 pb). (**D**) Amplicons for *tet*(H) (326 bp), *tet*(G) (460 bp), and *tet*(C) (629 bp). Water was used as a negative control.

Based on the discovery of pAsa8 and the presence of the *tet*(G) genes in this plasmid which was detected for the first time in *A. salmonicida* subsp *salmonicida*, primers targeting this gene were included in the fourth multiplex PCR assay (the one detecting *tet*(C) and *tet*(H)) (see the Methods section). An illustration of the results obtained with positive controls with all the four multiplex PCR assays designed in this study is shown in Fig. 3.

In addition, other primers were designed to target the various parts of pAsa8 (Supplementary Table S1). Three targets were for the pAsa8 backbone and one for the Tn*1721*. The primers targeting *tet*(G) were used to detect the presence of the In104-like since this gene was actually only found to be In104-encoded in *A. salmonicida* subsp. *salmonicida*. Only two isolates, M15448-11 and M16474-11, gave PCR-positive results with all the primer pairs tested. No amplification occurred with any of the primer pairs in any other isolates.

### Geographic distribution of isolates in Quebec harboring antibiotic resistance plasmids

**Figure 4:**
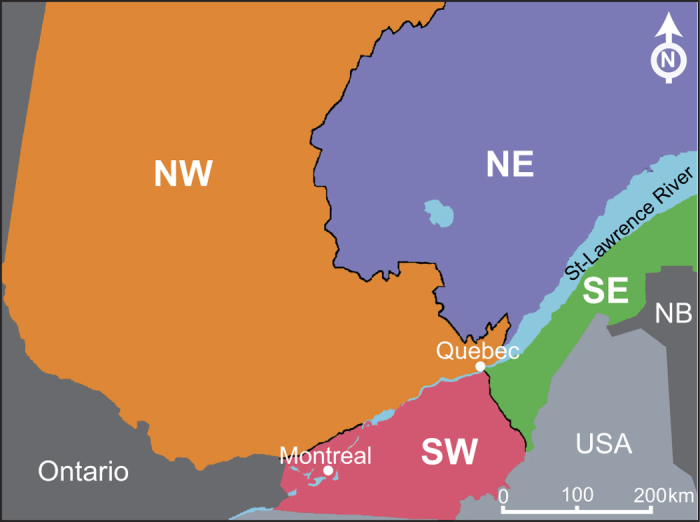
Map of the Quebec showing the regions to which the various isolates were assigned. Northwest (NW, orange), northeast (NE, purple), southwest (SW, pink), and southeast (SE, green). Most of the fish farms analyzed were less than 200 km from the St. Lawrence River, which crosses the province from west to east. The map has been drawn using Adobe Photoshop CS4 version 11.0.2 (www.adobe.com).

Since a large number of isolates from Quebec were included in the present study, it was relevant to analyze the geographical distribution of the antibiotic-resistant isolates in Quebec. The province was divided into four regions: southeast (SE), southwest (SW), northeast (NE), and northwest (NW) (Fig. 4 and Table 2). The multidrug-resistant plasmid pSN254b was identified in many isolates from the SE, SW, and NW regions, while pAsa4 variants were only found in three isolates from the NW region. The two isolates containing pAsa8 were also from the NW region. The isolates from the SE, SW and NW regions displayed a high prevalence of antibiotic resistance while those from the NE region displayed no antibiotic resistance at all.

## Discussion

One of the goals of this study was to evaluate the occurrence of genes in the fish pathogen *A. salmonicida* subsp. *salmonicida* that code for resistance to chloramphenicol/florfenicol, tetracycline, and sulfamethoxazole. Based on the multiplex PCR results, sulfonamide resistance was the most common antibiotic resistance detected in the Canadian isolates. The prevalence of florfenicol resistance in Quebec is most likely due to the intensive use of this antibiotic since 1999 to treat furunculosis^3^. The selective pressure caused by exposure to this antibiotic has promoted the spread of florfenicol-resistant isolates^20^. The multiplex PCR results showed that many isolates from Quebec contain resistance genes to the four antibiotics authorized for aquaculture use in the province. This is alarming since the Veterinary Drugs Directorate (VDD) of Health Canada has not authorized the use of any other antibiotics for treating fish infected by these multi-resistant isolates. However, given that the present study mainly investigated *A. salmonicida* subsp. *salmonicida* isolates from Quebec, more isolates from other Canadian provinces will have to be assessed to determine whether they also display the same trends.

The multiplex PCR-based method that we developed provide an accurate diagnostic tool allowing to identify genes on known plasmids in *A. salmonicida* subsp. *salmonicida* that code for resistance against the three major classes of antibiotics legally used in fish farming in Canada. While the present study focused only on *A. salmonicida* subsp. *salmonicida* isolates from Canada, this new diagnostic tool allowed us to uncover important information about the antibiotic resistance of this bacterium. This diagnostic method could also be used to study the diversity of antibiotic resistance plasmids in *A. salmonicida* subsp. *salmonicida* isolates from around the world and for conducting surveys to study temporal resistance evolution in general.

Since the discovery of transferable R-factors in *A. salmonicida* published in 1971 by Aoki^21^, many R-plasmids have been found in this bacterium (Table 1). However, actually only pSN254b (25/100), pRAS3 (4/100), pAsa4-like (4/100), pAB5S9b (2/100) and pAsa8 (2/100) were found in Canadian isolates. More specifically, the pSN254b multiple antibiotic resistance-encoding plasmid has been observed in many isolates in Quebec and is a major problem when it comes to the treatment of infected fish with antibiotics. The high prevalence of pSN254b can be mostly explained by (1) its capacity to be transferred by conjugation^22^ and (2) the presence of genes conferring resistance to various antibiotics, thus enhancing the selection pressure for isolates having this plasmid. In fact, up to 40% of the *A. salmonicida* subsp. *salmonicida* bacteria isolated from diseased fish in Quebec, except in the NE region, harbor antibiotic resistance genes (Table 2). pAsa4 and pAsa8 were found in the NW region while the pSN254b plasmid was present in the SW, SE, and NW regions.

The veterinarians who collected the *A. salmonicida* subsp. *salmonicida* isolates from diseased fish proposed three reasons explaining the geographic distribution of the antibiotic resistant isolates^23^. First, there is more infection outbreak in regions with higher aquaculture activity (mainly NW, SW and SE). Second, it is known that fish farmers exchange fish between regions which may contribute in part to the spread of the antibiotic resistant isolates. Finally, there is an increased likelihood of diagnosis for the fish farms in the surrounding regions of the veterinary service (e.g. NW and SW).

As mentioned in Vincent *et al*. (2014), plasmids conferring resistance to antibiotics may be transferable between *A. salmonicida* subsp. *salmonicida* and human pathogens such as *Salmonella enterica*^5^. This is exemplified by the IncA/C pSN254b plasmid, a variant of pSN254 found in *S. enterica*^5^. The plasmids of the IncA/C group are known to be conjugative and found, in addition to *A. salmonicida* and *S. enterica*, in a broad range of bacterium^24^ such as *Yersinia pestis*^25^, *Klebsiella pneumonia*^26^, *Aeromonas hydrophila*^27^,^28^, *Photobacterium damselae* subsp. *piscicida*^29^ and *Escherichia coli*^30^,^31^. The IncA/C plasmids are also known to regulate the excision of SGI1^32^ and to serve as helpers for its mobilization in *trans*^33^. Interestingly, pAsa8 (a new R-plasmid described here) of the M16474-11 and M15448-11 *A. salmonicida* subsp. *salmonicida* isolates appeared to have a Tn*1721* and a complex class 1 integron similar to the one found in SGI1. Even if we have a putative mechanism explaining the multiple integration events in the ancestral pAsa8 plasmid (Fig. 1), it is unclear if they occurred in *A. salmonicida*, in other bacteria such as *S. enterica*, or in both.

In summary, we analyzed 100 *A. salmonicida* subsp. *salmonicida* Canadian isolates for their antibiotic resistance genes and R-plasmids repertoire to shed light on their occurrence and distribution. We found that the conjugative R-plasmid pSN254b is dominant with 25% of the isolates having it. In fact, 37% of the isolates have at least one R-plasmid. This situation is worrying considering that no government-approved antibiotics can be used against these isolates to prevent furunculosis. We suggest that *A. salmonicida* subsp. *salmonicida* should be monitored worldwide to verify the trend found in Canada revealed in this study. We also discovered and characterized a new R-plasmid named here pAsa8. This plasmid hosts multiple antibiotic resistance genes from mobile elements such as Tn*1721* and a complex class 1 integron In104-like. Since In104 is usually found within the genomic island SGI1 of the human pathogen *S. enterica*, this study reinforces the idea that *A. salmonicida* subsp. *salmonicida* could be an important reservoir of mobile DNA conferring drug resistance and raises the possibility of exchange of this genetic material with other waterborne bacteria. If indeed this is the case, it is even more worrying from a One Health perspective (i.e. the idea that human, animal and environmental health are all interconnected)^34^. Considering antimicrobial resistance in a One Health context is a part of the fourth intervention plan of the last report produced by the *Review on Antimicrobial Resistance*^35^. It is now well known that environmental bacteria, such as *Aeromonas*^36^, may act as a reservoir of antibiotic resistance genes^37^,^38^,^39^. Therefore, our results add substantial evidence that there is an urgent need to develop alternative strategies to efficiently mitigate furunculosis prevalence without selecting for other resistance genes. Pre and probiotic strategies, phage therapy and vegetable extracts such as essential oils might be promising research avenues against *A. salmonicida* subsp. *salmonicida* as it is the case for other bacteria^40^,^41^,^42^.

## Methods

### Bacterial isolates and growth conditions

All 100 *A. salmonicida* subsp. *salmonicida* isolates used in this study were from Canada (Supplementary Table S2). The A449 reference strain from France^43^, for which the annotated chromosome and plasmid sequences are publicly available, was also included. All the isolates were grown on furunculosis agar for two or three days at 18 °C as previously described^44^.

### PCR analyses

The DNA templates, PCR mixtures, and program cycles used in this study have been previously described^45^. The PCR assays were performed at least twice. Suitable positive and negative controls were included in each assay.

The PCR primers used to identify the isolates harboring antibiotic-resistance plasmids (pAsa4, pAB5S9, pSN254, pRAS3) are listed in Supplementary Table S1, as are the primers used to genotype pAsa8 of the M16474-11 isolate.

### Multiplex PCR

The sequences of orthologous genes causing resistance to chloramphenicol/florfenicol (*cat* and *floR*), tetracycline (*tetA*, *tet*(C), *tet*(E), *tet*(H), *tet*(G) and sulfamethoxazole (*sul1* and *sul2*) in *A. salmonicida* subsp. *salmonicida* were aligned to identify the conserved regions. The chloramphenicol resistance gene *catA2* found on the pAr-32 plasmid identified in a Japanese isolate^46^ was not included in the analysis due to the lack of an appropriate positive control. The alignment for each resistance gene sequence was performed using MUSCLE^47^ and BLASTn^48^ through Geneious version 6.1.8^49^. Primers were designed based on the conserved sequences using Primer3^50^ also through Geneious version 6.1.8.

The DNA templates were prepared by resuspending an inoculum of bacterial culture in 1 mL of SWL buffer (50 mM KCl, 10 mM Tris, pH 8.3, 2.5 mM MgCl_2_, 0.45% NP-40, and 0.45% Tween 20). The lysates were heated for 15 min at 95 °C, and the DNA concentrations in the solutions were adjusted to 100 ng/μL. The PCR mixture contained 1X Go-Taq buffer (Promega, USA), 2 mM dNTP, 0.2 μM forward and reverse primers, 1 U of GoTaq (5 U; Promega), and 100 ng of DNA templates. Depending on the number of primers used for the multiplex PCR, various volumes of filtered water were added to adjust the final reaction volume to 20 μL. The PCR program was as follows: 2 min 30 s at 95 °C, 30 cycles of 30 s at 95 °C, 30 s at 60 °C, and 1 min 30 s at 68 °C, followed by a final 5-min extension step at 68 °C. The PCR products were separated on 1.3% agarose gels and were stained with 0.5 μg/mL of ethidium bromide. For all the multiplex PCR, water and 01-B526, an antibiotic-sensitive isolate^51^, were used as negative controls. The primers used for each multiplex PCR are given in Table 3.

In the first multiplex PCR, targeting *floR* and *cat*, a mix of the sequenced isolates M15879-11 and A449^43^,^52^ was used as a positive control. The *floR* gene gives resistance to both chloramphenicol and florfenicol while the *cat* gene provides resistance only to chloramphenicol^53^. Bioinformatics analyses confirmed that the M15879-11 isolate bears a pSN254b plasmid similar to that of the 2004-05MF26 isolate and had the same set of antibiotic resistance genes^5^,^54^. In the second multiplex PCR, developed to detect the *sul1* and *sul2* genes, the M15879-11 isolate was used as positive control. A subset of tetracycline resistance genes (*tetA* and *tet*(E)) was targeted by a third multiplex PCR. A mix of M15879-11 and A449 was used as a positive control for these genes. The last multiplex PCR assay aimed at detecting *tet*(C), *tet*(G) and *tet*(H). Two isolates were used as positive controls: 2009-144K3 (pAB5S9b and pRAS3.3) and M16474-11 (pAsa8).

### DNA extraction and genomic sequencing

The total DNA of the M15879-11 and M16474-11 *A. salmonicida* subsp. *salmonicida* isolates was extracted using DNeasy Blood and Tissue kits (Qiagen, Canada) and was sequenced by next-generation sequencing (NGS) at the Plateforme d’Analyse Génomique of the Institut de Biologie Intégrative et des Systèmes (IBIS, Université Laval). The DNA of the M15879-11 isolate was fragmented at a 5-kb size and sequenced by pyrosequencing using a GS-FLX+ apparatus as previously described^52^. The resulting sequencing reads were *de novo* assembled with Newbler version 2.5.3^55^. The total draft assembly of M15879-11 was annotated by the NCBI Prokaryotic Genome Annotation Pipeline (PGAP) and deposited in GenBank under accession number LAIS00000000.

The M16474-11 library was prepared using a KAPA Hyper Prep kit and was sequenced by a MiSeq (Illumina) sequencing system. The resulting reads were filtered with Trimmomatic version 0.32^56^ using the manual-recommended parameters for paired-end reads^57^. The DNA of M16474-11 was also extracted by phenol/chloroform by following the protocol *Extracting DNA Using Phenol-Chloroform* provided by Pacific Biosciences (http://www.pacb.com). The DNA was sheared at 8-kb using a standard Covaris g-TUBE protocol and was used to prepare an Oxford nanopore technologies sequencing library using the protocol GDE_1002_v1_revF_17Nov2015. The library was sequenced with a MinION sequencer. The basecalling was done by the Oxford nanopore technologies’s Metrichor cloud service with the *2D Basecalling for SQK-MAP006* version 1.69 application. All the reads (1D and 2D) ≥ 750 bp were converted from the native HDF5/FAST5 format to FASTQ by poretools version 0.5.1^58^. Finally, a hybrid assembly of both Illumina and Oxford nanopore technologies reads was performed using SPAdes version 3.7.1^59^ with kmer lengths of 21, 33, 55, 77, 99 and 127. The pAsa8 plasmid was recovered in a single contig. To assess the quality of the sequence, the Illumina reads were mapped on the pAsa8 sequence using BWA-MEM^60^ and evaluated with Pilon version 1.17^61^. No corrections occurred during step, reinforcing our confidence in the quality of the pAsa8 sequence. This sequence was annotated with the webserver RAST^62^ and manually curated. The sequence was analyzed using TAfinder (TADB) to find putative type 2 toxin-antitoxin systems^63^. The complete annotated sequence of pAsa8 was deposited in GenBank under the accession number KX364409.

## Additional Information

**How to cite this article**: Trudel, M. V. *et al*. Diversity of antibiotic-resistance genes in Canadian isolates of *Aeromonas salmonicida* subsp. *salmonicida*: dominance of pSN254b and discovery of pAsa8. *Sci. Rep.* **6**, 35617; doi: 10.1038/srep35617 (2016).

## Supplementary Material

Supplementary Information

## Figures and Tables

**Table 1:** R-plasmids found in *A. salmonicida*.

Plasmid	Length (kb)	R-genes	Found in Canada	Reference
pAsa7	5,276	*cat*	No	64
pRAS3.2	11,823	*tetA*	No	65
pRAS3.3	11,845	*tetA*	Yes	5
pRAS3.1	11,851	*tetA*	No	65
pAB5S9b	25,540	*tet*(H)*, floR, sul2, strA, strB*	Yes	5
pASOT3	~39	*aadA2, tetA*	No	66,67
pRAS1	~45	*dfrA16, tetA, sul1*	No	17
pAr-32	~47	*aadA2, sul1, catA2*	No	17,21
pASOT	~47	*aadA2 or dfrIIc, tetA*	No	66,67
pASOT2	~47	*aadA2, tetA*	No	66,67
pRAS2	~48	*sul2, tet 31, strA, strB*	No	17,68
pSN254b	152,216	*tetA, floR, sul1, sul2, blaCMY, aadA, strA, strB, sugE2, qacEΔ1*	Yes	5
pAsa4	166,749	*tet*(E)*, sul1, aadA, cat*	Yes (pAsa4-like)	5,43

**Table 2:** Geographical distribution of the antibiotic resistant isolates in Quebec.

Region^a^		NE	SE	SW	NW	Quebec as a whole
Number of isolates		16	11	14	45	86
Plasmid found in the isolates	pSN254b	0	5	6	12	23
	pAsa4-like	0	0	0	3	3
	pAsa4	0	0	0	0	0
	pAB5S9	0	0	0	0	0
	pRAS3	0	0	0	0	0
	pAsa8	0	0	0	2	2
Number of antibiotic-resistant isolates		0	5	6	17	28
% of resistant isolates^b^		0 [0.00, 0.11]	45 [0.20, 0.73]	43 [0.20, 0.68]	38 [0.25, 0.52]	33 [0.23, 0.43]

^a^see Fig. 4.

^b^The binomial 95% confidence interval (Jeffreys method) is indicated between brackets.

**Table 3:** Primers used for the multiplex PCR.

Primer	Sequence 5′−3′	Tm (°C)	Amplicon size (bp)	Target
Multiplex PCR targeting genes coding for chloramphenicol/florfenicol resistance				
MT-ctrl-chloram-F1	GCTTACCTCAGATAATGAGTCGTC	54,8	172	Prophage 1 (Control)
MT-ctrl-chloram-R1	GCCAATAAGAGCCCTACTCTTC	55		
MT-cat-F1	CTATTTTGACAATACGCCCTGC	54,3	448	*cat*
MT-cat-R1	CTTCCCAAACGTAAATATCGGC	54		
MT-floR-F1	TTGAGCCTCTATATGGTGATGC	54,4	632	*floR*
MT-floR-R1	GTTGTCACGATCATTACAAGCG	54,3		
Multiplex PCR targeting genes coding for sulfonamide resistance				
MT-ctrl_sul-F1	TTCATTTCGTCTTGGGTCTAGC	54,8	175	Prophage 1 (Control)
MT-ctrl_sul-R1	GGACTACAGATCTACCATAATCCG	54		
MT-sul1-F1	GGGCTACCTGAACGATATCC	54,7	550	*sul1*
MT-sul1-R1	CTAGGCATGATCTAACCCTCG	54,4		
MT-sul2-F1	ATCATCTGCCAAACTCGTCG	55,2	449	*sul2*
MT-sul2-R1	TTCTTGCGGTTTCTTTCAGC	53,9		
Multiplex PCR targeting genes coding for tetracycline resistance				
First multiplex PCR targeting genes coding for tetracycline resistance				
MT-ctrltet1-F1	CCAGAATGACGAATTGAATGTCG	54,3	175	Prophage 1 (Control)
MT-ctrltet1-R1	GGACCTCTTTACTCCAGTCG	54,4		
MT-tetA(E)pAsa4-F1	GATGTCACACCTGAGGAATCC	55,1	351	*tetA*(E)
MT-tetA(E)pAsa4-R1	TCCGAATAAAACCCATAATGTTGC	53,9		
MT-tetApSN54b-F1	CAAGCAGGATGTAGCCTGTG	55,9	526	*tetA*
MT-tetApSN54b-R1	ATTGCCGATATCACTGATGG	52,4		
Second multiplex PCR targeting genes coding for tetracycline resistance				
MT-ctrltet2-F1	ATTCATTTCGTCTTGGGTCTAGC	55	176	Prophage 1 (Control)
MT-ctrltet2-R1	GGACTACAGATCTACCATAATCCG	54		
MT-tetHpAB5S9b-F1	ACGACTGTCTGATAAATACGGC	54,6	326	*tetH*
MT-tetHpAB5S9b-R1	ATATCGAGTGTGAAATAGCGGC	54,9		
MT-tetA(C)pRAS3-F1	CTGTAGGCATAGGCTTGGTTAT	54,4	629	*tetA*(C)
MT-tetA(C)pRAS3-R1	CTGTCCTACGAGTTGCATGATA	54,1		
MT-tetGs62-F1	GGTTCGCATCAAACCATTCG	54,8	460	tetA(G)
MT-tetGs62-R1	GCTTAGATTGGTGAGGCTCG	55,6		
