# Supplementary material for: Diversity of antibiotic-resistance genes in Canadian isolates of Aeromonas salmonicida subsp. salmonicida: dominance of pSN254b and discovery of pAsa8
Source: Sci Rep. 2016 Oct 18;6:35617. doi: 10.1038/srep35617 (PMC5067588; doi:10.1038/srep35617)
Supplement: Supplementary Information [file srep35617-s1.pdf]

Diversity of antibiotic-resistance genes in Canadian isolates of *Aeromonas salmonicida* subsp. *salmonicida*: dominance of pSN254b and discovery of pAsa8

Mélanie V. Trudel[1,2,3*], Antony T. Vincent[1,2,3*], Sabrina A. Attéré[1,2,3], Myriam Labbé[1,2,4], Nicolas Derome[1,5], Alexander I. Culley[1,2,4] and Steve J. Charette[1,2,3,#]

1. Institut de Biologie Intégrative et des Systèmes (IBIS), Université Laval, Quebec City, QC, Canada, G1V 0A6

2. Département de biochimie, de microbiologie et de bio-informatique, Faculté des sciences et de génie, Université Laval, Quebec City, QC, Canada, G1V 0A6

3. Centre de recherche de l'Institut universitaire de cardiologie et de pneumologie de Québec (IUCPQ), Quebec City, QC, Canada, G1V 4G5

4. Groupe de Recherche en Écologie Buccale (GREB), Faculté de médecine dentaire, Université Laval, Quebec City, QC, Canada, G1V 0A6

5. Département de biologie, Faculté des sciences et de génie, Université Laval, Quebec City, QC, Canada, G1V 0A6

[#]To whom correspondence should be addressed: Institut de Biologie Intégrative et des Systèmes (IBIS), Pavillon Charles-Eugène-Marchand, 1030 avenue de la Médecine, Université Laval, Quebec City, QC, Canada, G1V 0A6

steve.charette@bcm.ulaval.ca; Telephone: 1-418-656-2131, ext. 6914; Fax: 1-418-656-7176

[*]These authors contributed equally to this work.

Running title: *A. salmonicida*: pSN254b dominance and pAsa8 discovery

Supplementary Table S1. Primers used in this study

Primer	Sequence (5'-3')	Reference
**PCR positive control (*tapA* gene on the chromosome)**		
tapA F	ACATGAAGAAGCAATCAGGC	[1]
tapA R	AGAGGTCATGCGTTAGCAG	[1]
**PCR for the detection of pAB5S9**		
ORF101-strB_F1[a]	CTCGTGAATGTCGGTATCCTGTCT	[2]
ORF101-strB_R1[a]	CTCGGTCGTGAGAACAATCTGATG	[2]
parA-resA_F1	CGTCAATGATGATGTGGTCGTACC	[2]
parA-resA_R1	CTGATCGACCAGGAAGGCTATAGC	[2]
ORF397-tnp_F1	TGTTAGCACTCGGTTGCCTTCGAT	[2]
ORF397-tnp_R1	GACATACACACCGTCGAGAAACAG	[2]
**PCR for the detection of pSN254**		
tnp-urf2_F1[b]	CATTTCGGTCGACCATACGACGATC	[2]
tnp-urf2_R1[b]	CTGCGTGTGCTGCAAGGAAATCC	[2]
traC_F1	CTACGACAATGGCTTGATCCAAGAC	[2]
traC_R1	GTACAGAATGGCAGGCAGTTGAGG	[2]
ORF1417_F1	CGAGACCTTCCCTTTTACTGATGC	[2]
ORF1417_R1	CAGACAGAGGACGAGTTGTAGAAC	[2]
**PCR for the detection of pAsa4**		
DD1-pAsa4-traG-F1	AGGTTGCTCTGGAAAGCCTCTGAT	[2]
DD2-pAsa4-traG-R1	TGTGGATGCCTGTGCTCTCCATTA	[2]
**PCR for the characterization of pRAS3**		
Reg_1_F2	GGATAGTCGATCTGCTGGATACG	[2]

Reg_1_R2	CTACCCTGTGGAACACCTACATCT	[2]
Reg_2_F1	CGTTGTGGATGTGCTTCAGCAATC	[2]
Reg_2_R1	GAGTGGTTGAGCAATACAGGATGC	[2]
**PCR for the characterization of pAsa8**		
Geno_pAsa8_F1b	CATGATGGTCACACCTCGATACTC	This study
Geno_pAsa8_R1	GCAATCCACTACTCAGTAGGTGAG	This study
Geno_pAsa8_F2b	GGAATCTGGTGGATGCGCATCTTC	This study
Geno_pAsa8_R2	CGTCTGATCCAAGAACAACCATCC	This study
Geno_pAsa8_F4	GTGATGATGTGAAATCTGCGTGGG	This study
Geno_pAsa8_R4	GCACTATCGCTTGCTTTCACCAGG	This study
Geno_pAsa8_F5	GCTTGCGGTCTACAGTGATATCTC	This study
Geno_pAsa8_R5	CGGTACTCAAATGCAACGCTACTAC	This study

a. The ORF101-strB primers were designed to give different amplicon sizes in pAB5S9 and pAB5S9b.
b. The tnp-urf2 primers were specific to pSN254b.

References

1 Ebanks, R. O. *et al.* Expression of and secretion through the *Aeromonas salmonicida* type III secretion system. *Microbiology* **152**, 1275-1286, doi:152/5/1275 [pii]10.1099/mic.0.28485-0 (2006).

2 Vincent, A. T. *et al.* Detection of variants of the pRAS3, pAB5S9, and pSN254 plasmids in *Aeromonas salmonicida* subsp. *salmonicida*: multidrug resistance, interspecies exchanges, and plasmid reshaping. *Antimicrob Agents Chemother* **58**, 7367-7374, doi:10.1128/AAC.03730-14 (2014).

Supplementary Table S2. *A. salmonicida* subsp. *salmonicida* isolates used in this study

Isolate	Origin			Date of isolation	Plasmids found by PCR genotyping	Genes found by multiplex PCR	Plasmids[b]					Prophages[c]			*AsaGEI*[c]	Phenotypic assay[d]		
	Fish	Region[a]	Quebec subregion				pAsa1	pAsa2	pAsa3	pAsal1	pAsa5	1	2	3		A-layer	Lipases	Proteases
A449	Brown trout	France		1975	pAsa4	*cat, sul1, tetA*(E)	+	+	+	None	+	+	+	+	None	+	+	-
01-B522	Brook trout	Quebec (NW)	NW-B	2001	pAsa4-like (*traG*+)	*sul1, tetA*(E)	+	+	+	+	+	+	+	+	1a	+	+	+
01-B526	Brook trout	Quebec (NW)	NW-C	2001	-	-	+	+	+	+	+	+	+	+	1a	+	+	+
01-B516	Brook trout	Quebec (NW)	NW-H	2001	-	-	+	+	+	+	+	+	+	+	2a	+	+	+
07-9324	Brook trout	Quebec (NW)	NW-B	2007	pSN254b	*floR, sul1, sul2, tetA*	+	+	+	+	+	+	+	+	1a	+	+	+
07-7817	N/A[e]	Quebec (NW)	NW-B	2007	pSN254b	*floR, sul1, sul2, tetA*	+	+	+	+	+	+	+	+	1a	+	+	+
07-7346	Atlantic salmon	Quebec (NE)	NE-D	2007	-	-	+	+	+	+	+	+	+	None	2a	+	+	+
07-5957	Atlantic salmon	Quebec (NE)	NE-D	2007	-	-	+	+	+	+	+	+	+	None	2a	+	+	+
07-7287	Brook trout	Quebec (SW)	SW-C	2007	pSN254b	*floR, sul1, sul2, tetA*	+	+	+	+	+	+	+	+	1a	+	+	+
08-2647	Brook trout	Quebec (NW)	NW-B	2008	pSN254b	*floR, sul1, sul2, tetA*	+	+	+	+	+	+	+	+	1a	+	+	+
08-2783	Brook trout	Quebec (SW)	SW-C	2008	pSN254b	*floR, sul1, sul2, tetA*	+	+	+	+	+	+	+	+	1a	+	+	+
08-4188	Brook trout	Quebec (NW)	NW-B	2008	pSN254b	*floR, sul1, sul2, tetA*	+	+	+	+	+	+	+	+	1a	+	+	+
09-0167	Atlantic salmon	Quebec (NE)	NE-D	2009	-	-	+	+	+	+	+	+	+	None	2a	+	+	+
m17524-09	Brook trout	Quebec (NW)	NW-B	7-Sep-09	-	-	+	+	+	+	+	+	+	+	2a	+	+	+
m14349-09	Atlantic salmon	Quebec (NW)	NW-B	04-aout-09	pSN254b	*floR, sul1, sul2, tetA*	+	+	+	+	+	+	+	+	1a	+	+	+

m23281-09	Brook trout	Quebec (NW)	NW-G	03-dec-09	-	-	+	+	+	+	+	+	+	+	2a	+	+	+
m23067-09	Brook trout	Quebec (NE)	NE-C	01-dec-09	-	-	+	+	+	+	+	+	+	+	2a	+	+	+
m19438-09	Brook trout	Quebec (NW)	NW-B	13-Oct-09	-	-	+	+	+	+	+	+	+	+	2a	+	+	+
m16583-09	Brook trout	Quebec (NW)	NW-B	2-Sep-09	-	-	+	+	+	+	+	+	+	+	2a	+	+	+
m14231-09	Atlantic salmon	Quebec (NE)	NE-D	13-Jul-09	-	-	+	+	+	+	+	+	+	+	2a	+	+	+
m11743-09	Brook trout	Quebec (SW)	SW-C	2-Jul-09	-	-	+	+	+	+	+	+	+	+	2a	+	+	+
m11431-09	Brook trout	Quebec (SW)	SW-C	30-Jun-09	-	-	+	+	+	+	+	+	+	+	2a	+	+	+
m10419-09	Brook trout	Quebec (SW)	SW-C	17-Jun-09	-	-	+	+	+	+	+	+	+	+	2a	+	+	+
m9906-09	Brook trout	Quebec (SW)	SW-C	11-Jun-09	pSN254b	*floR, sul1, sul2, tetA*	+	+	+	+	+	+	+	+	1a	+	+	+
m9954-10	Brook trout	Quebec (NW)	NW-B	20-May-10	-	-	+	+	+	+	+	+	+	+	1a	+	+	+
m8029-10	Brook trout	Quebec (NW)	NW-B	28-Apr-10	-	-	+	+	+	+	+	+	+	+	2a	+	+	+
m11603-10	Brook trout	Quebec (NW)	NW-B	10-Jun-10	-	-	+	+	+	+	+	+	+	+	2a	+	+	+
m6363-10	Brook trout	Quebec (NE)	NE-C	7-Apr-10	-	-	+	+	+	+	+	+	+	+	2a	+	+	+
m9221-10	Brook trout	Quebec (NE)	NE-C	11-May-10	-	-	+	+	+	+	+	+	+	+	2a	+	+	+
M10935-11	Brook trout	Quebec (NW)	NW-B	2011	-	-	+	+	+	+	+	+	+	+	2a	+	+	+
M15448-11	Brook trout	Quebec (NW)	NW-B	2011	pAsa8	*floR, sul1, tetG, tetA*	+	+	+	+	+	+	+	+	2a	+	+	+
M16474-11	Brook trout	Quebec (NW)	NW-B	2011	pAsa8	*floR, sul1, tetG, tetA*	+	+	+	+	+	+	+	+	2a	+	+	+
M19878-11	Brook trout	Quebec (NW)	NW-B	2011	-	-	+	+	+	+	+	+	+	+	2a	+	+	+
M11500-11	Brook trout	Quebec (NW)	NW-H	2011	-	-	+	+	+	+	+	+	+	+	2a	+	+	+

M16486-11	Brook trout	Quebec (NW)	NW-H	2011	-	-	+	+	+	+	+	+	+	+	2a	+	+	+
M13460-11	Brook trout	Quebec (SE)	SE-A	2011	-	-	+	+	+	+	+	+	+	+	2a	+	+	+
M13729-11	Brook trout	Quebec (SE)	SE-A	2011	-	-	+	+	+	+	+	+	+	+	2a	+	+	+
M14481-11	Brook trout	Quebec (SE)	SE-B	2011	pSN254b	*floR, sul1, sul2, tetA*	+	+	+	+	+	+	+	+	1a	+	+	+
M15879-11	Brook trout	Quebec (SE)	SE-B	2011	pSN254b	*floR, sul1, sul2, tetA*	+	+	+	+	+	+	+	+	1a	+	+	+
M17739-11	Brook trout	Quebec (SE)	SE-B	2011	pSN254b	*floR, sul1, sul2, tetA*	+	+	+	+	+	+	+	+	1a	+	+	+
M13732-11	Brook trout	Quebec (SW)	SW-C	2011	pSN254b	*floR, sul1, sul2, tetA*	+	+	+	+	+	+	+	+	1a	+	+	+
M17053-11	Brook trout	Quebec (SW)	SW-C	2011	pSN254b	*floR, sul1, sul2, tetA*	+	+	+	+	+	+	+	+	1a	+	+	+
M15878-11	Rainbow trout	Quebec (SE)	SE-B	2011	-	-	+	+	+	+	+	+	+	+	2a	+	+	+
M13182-11	Atlantic salmon	Quebec (NE)	NE-C	2011	-	-	+	+	+	+	+	+	+	+	2a	+	+	+
M17735-11	Brook trout	Quebec (NE)	NE-C	2011	-	-	+	+	+	+	+	+	+	+	2a	+	+	+
M15576-11	Brown trout	Quebec (NW)	NW-B	2011	-	-	+	+	+	+	+	+	+	None	2a	+	+	+
M15469-11	Brook trout	Quebec (NW)	NW-B	2011	pSN254b	*floR, sul1, sul2, tetA*	**N/A**	**N/A**	**N/A**	**N/A**	**N/A**	+	+	+	1a	+	+	+
M22710-11	Brook trout	Quebec (SW)	SW-C	2011	-	-	+	+	+	+	+	+	+	+	2a	+	+	+
M13764-11	Brook trout	Quebec (NW)	NW-G	2011	-	-	+	+	+	+	+	+	+	+	2a	+	+	+
M18076-11	Lumpfish	Quebec (SE)	SE-A	2011	-	-	+	+	+	+	+	+	+	+	None	+	+	+
M23911-11	Brook trout	Quebec (NW)	NW-B	2011	-	-	+	+	+	+	+	+	+	+	1a	+	+	+
M12357-12	Brook trout	Quebec (NW)	NW-B	12-Jun-12	pSN254b	*floR, sul1, sul2, tetA*	+	+	+	+	+	+	+	+	1a	+	+	+
M21375-12	Brook trout	Quebec (NE)	NE-C	4-Oct-12	-	-	+	+	+	+	+	+	+	+	2a	+	+	+

M16237-12	Brook trout	Quebec (NE)	NE-C	1-Aug-12	-	-	+	+	+	+	+	+	+	+	2a	+	+	+
M12976-12	Brook trout	Quebec (NW)	NW-H	19-Jun-12	-	-	+	+	+	+	+	+	+	+	2a	+	+	+
M22895-12	Brook trout	Quebec (NW)	NW-G	25-Oct-12	-	-	+	+	+	+	+	+	+	+	2a	+	+	+
M10745-12	Brown trout	Quebec (NW)	NW-B	4-May-12	pSN254b	*floR, sul1, sul2, tetA*	+	+	+	+	+	+	+	+	1a	+	+	+
M9754-12	Brook trout	Quebec (NW)	NW-G	11-May-12	-	-	+	+	+	+	+	+	+	+	2a	+	+	+
M17930-12	Brook trout	Quebec (SW)	SW-C	23-Aug-12	-	-	+	+	+	+	+	+	+	+	1a	+	+	+
M12418-12	Brook trout	Quebec (NW)	NW-B	13-Jun-12	-	-	+	+	+	+	+	+	+	+	2a	+	+	+
M21368-12	Brook trout	Quebec (NW)	NW-C	4-Oct-12	-	-	+	+	+	+	+	+	+	+	2a	+	+	+
M13050-12	Brook trout	Quebec (NW)	NW-H	27-Jun-12	pSN254b	*floR, sul1, sul2, tetA*	+	+	+	+	+	+	+	+	2a	+	+	+
M13566-12	Brook trout	Quebec (SE)	SE-B	27-Jun-12	pSN254b	*floR, sul1, sul2, tetA*	+	+	+	+	+	+	+	+	1a	+	+	+
M14404-12	Atlantic salmon	Quebec (NE)	NE-C	10-Jul-12	-	-	+	+	+	+	+	+	+	+	2a	+	+	+
M16671-12	Brook trout	Quebec (NW)	NW-H	7-Aug-12	-	-	+	+	+	+	+	+	+	+	2a	+	+	+
M16042-12	Brook trout	Quebec (SE)	SE-B	30-Jul-12	pSN254b	*floR, sul1, sul2, tetA*	+	+	+	+	+	+	+	+	1a	+	+	+
SHY13-162	Brook trout	Quebec (NE)	NE-C	25-Jan-13	-	-	+	+	+	+	+	+	+	+	2a	+	+	+
SHY13-574	Brook trout	Quebec (NE)	NE-C	25-Feb-13	-	-	+	+	+	+	+	+	+	+	2a	+	(+)	+
SHY13-1470	Brook trout	Quebec (NW)	NW-H	9-May-13	-	-	+	+	+	+	+	+	+	+	2a	+	+	+
SHY13-2188	Brook trout	Quebec (NW)	NW-B	28-Jun-13	pSN254b	*floR, sul1, sul2, tetA*	+	+	+	+	+	+	+	+	1a	+	+	+
SHY13-2222	Brook trout	Quebec (NE)	NE-C	3-Jul-13	-	-	+	+	+	+	+	+	+	+	2a	+	-	+
SHY13-2257	Brook trout	Quebec (NW)	NW-G	8-Jul-13	-	-	+	+	+	+	+	+	+	+	2a	+	+	+

SHY13-2263	Brook trout	Quebec (NW)	NW-H	8-Jul-13	-	-	+	+	+	+	+	+	+	+	2a	+	+	+
SHY13-2317	Brook trout	Quebec (SE)	SE-B	12-Jul-13	-	-	+	+	-	+	+	+	+	+	None	+	+	+
SHY13-2425	Brook trout	Quebec (SW)	SW-C	22-Jul-13	-	-	+	+	-	+	+	+	+	+	None	+	+	+
SHY13-2458	Brook trout	Quebec (NW)	NW-B	22-Jul-13	pSN254b	*floR, sul1, sul2, tetA*	+	+	+	+	+	+	+	+	1a	+	+	+
SHY13-2534	Atlantic salmon	Quebec (NE)	NE-D	31-Jul-13	-	-	+	+	+	+	+	+	+	None	2a	+	+	+
SHY13-2627	Brook trout	Quebec (NW)	NW-H	9-Aug-13	pAsa4-like (*traG*+)	*sul1, tetA*(E)	+	+	+	+	+	+	+	+	2a	+	+	+
SHY13-2630	Brook trout	Quebec (NW)	NW-B	9-Aug-13	pSN254b	*floR, sul1, sul2, tetA*	+	+	+	+	+	+	+	+	1a	+	+	+
SHY13-2825	Brook trout	Quebec (SE)	SE-B	20-Aug-13	-	-	+	+	+	+	+	+	+	+	None	+	-	+
SHY13-2873	Brook trout	Quebec (SW)	SW-C	22-Aug-13	-	-	+	+	+	+	+	+	+	+	None	+	+	+
SHY13-2909	Brook trout	Quebec (NW)	NW-H	27-Aug-13	-	-	+	+	+	+	+	+	+	+	2a	+	+	+
SHY13-3101	Brook trout	Quebec (SW)	SW-C	12-Sep-13	-	-	+	+	+	+	+	+	+	+	1a	+	+	+
SHY13-3127	Brook trout	Quebec (NW)	NW-B	12-Sep-13	-	-	+	+	+	+	+	+	+	+	2a	+	+	+
SHY13-3795	Brook trout	Quebec (SW)	SW-C	23-Oct-13	pSN254b	*floR, sul1, sul2, tetA*	+	+	+	-	+	+	+	+	1a	+	+	+
SHY13-3798	Brook trout	Quebec (NW)	NW-H	23-Oct-13	-	-	+	+	+	+	+	+	+	+	2a	+	+	+
SHY13-3799	Brook trout	Quebec (NW)	NW-H	23-Oct-13	pAsa4-like (*traG*+)	*sul1, tetA*(E)	+	+	+	+	+	+	+	+	2a	+	+	+
2004-05 MF26	N/A	New Brunswick		2004	pSN254b	*floR, suI1, sul2, tetA*	+	+	+	+	+	+	+	+	2a	+	+	+
2005-175 K2	Brook trout	New Brunswick		2005	-	-	+	+	+	+	+	+	+	+	2a	+	+	+
2009-178 K9	Atlantic salmon	New Brunswick		2009	-	-	+	+	+	+	+	+	+	+	2a	+	+	+

2009-157 K5	Brook trout	New Brunswick		2009	pRAS3	*tetA*(C)	+	+	+	+	+	+	+	+	2a	+	+	+
2009-195 K29	Brook trout	New Brunswick		2009	pRAS3	*tetA*(C)	+	+	+	+	+	+	+	+	2a	+	+	+
2009-144 K3	Brook trout	New Brunswick		2009	pAB5S9b, pRAS3	*floR, sul2, tetH, tetA*(C)	+	+	+	+	+	+	+	+	2a	+	+	+
2010-47 K18	Brook trout	New Brunswick		2010	pAB5S9b, pRAS3	*floR, sul2, tetH, tetA*(C)	+	+	+	+	+	+	+	+	2a	+	+	+
2004-68 K52	Atlantic salmon	Nova Scotia		2004	pSN254b	*floR, sul1, sul2, tetA*	+	+	+	+	+	+	+	+	2a	+	+	+
RS 1458	Rainbow trout	Ontario		1993	pAsa4-like (*traG*+)	*tetA*(E)	+	+	+	+	+	+	+	+	2a	+	+	+
RS 1706	Chinook salmon	Ontario		2000	-	-	+	+	+	+	+	+	+	+	2a	+	+	+
RS 1705	Brook trout	Ontario		2000	-	-	+	+	+	+	+	+	+	+	1a	+	+	+
RS 1744	Coho salmon	Ontario		2000	-	-	+	+	+	+	+	+	+	+	2a	+	+	+
RS 1752	Pumpkinseed	Ontario		2001	-	-	+	+	+	+	+	+	+	+	1b	+	+	+
RS 1835	Coho salmon	Ontario		2011	-	-	+	+	+	-	+	+	+	+	2a	+	+	+

a. NW = northwest, SW = southwest, NE = northeast, SE = southeast (see Figure 4).

b. See : Attere SA, et al. Diversity and Homogeneity among Small Plasmids of *Aeromonas salmonicida* subsp. *salmonicida* Linked with Geographical Origin. Front Microbiol 6: 1274. (2015)

c. See: Emond-Rheault JG, et al. Variants of a genomic island in *Aeromonas salmonicida* subsp. *salmonicida* link isolates with their geographical origins. Vet Microbiol 175: 68-76. (2015)

d. See: Daher, R. K. et al. Alteration of virulence factors and rearrangement of pAsa5 plasmid caused by the growth of *Aeromonas salmonicida* in stressful conditions. Vet Microbiol 152, 353-360 (2011).

e. N/A: Not available
